# A comparison of effects of lard and hydrogenated vegetable shortening on the development of high-fat diet-induced obesity in rats

**DOI:** 10.1038/nutd.2015.40

**Published:** 2015-12-14

**Authors:** R Kubant, A N Poon, D Sánchez-Hernández, A F Domenichiello, P S P Huot, E Pannia, C E Cho, S Hunschede, R P Bazinet, G H Anderson

**Affiliations:** 1 Department of Nutritional Sciences, Faculty of Medicine, University of Toronto, Toronto, Ontario, Canada; 2 Department of Physiology, Faculty of Medicine, University of Toronto, Toronto, Ontario, Canada

## Abstract

**Background:**

Obesity is associated with increased consumption and preference for dietary fat. Experimental models of fat-induced obesity use either lard or vegetable shortening. Yet, there are no direct comparisons of these commonly used fat sources, or the influence of their fatty acid composition, on the development of diet-induced obesity.

**Objective:**

To compare the effects of lard and hydrogenated vegetable-shortening diets, which differ in their fatty acid composition, on weight gain and the development of obesity and insulin resistance in rats.

**Methods and design:**

Male Wistar rats were fed *ad libitum* for 14 weeks high-fat diets containing either (1) high vegetable fat (HVF, 60 kcal% from vegetable shortening) or (2) high lard fat (HLF, 60 kcal% from lard). Rats fed normal-fat (NF, 16 kcal% from vegetable shortening) diet served as control. Body weight, food intake, adipose tissue mass, serum 25[OH]D_3_, glucose, insulin and fatty acid composition of diets were measured.

**Results:**

Rats fed either of the two high-fat diets had higher energy intake, weight gain and fat accretion than rats fed normal-fat diet. However, rats fed the HLF diet consumed more calories and gained more weight and body fat with greater increases of 32% in total (158.5±8.2 vs 120.2±6.6 g, *P<*0.05), 30% in visceral (104.4±5.2 vs 80.3±4.2 g, *P*<0.05) and 36% in subcutaneous fat mass (54.1±3.6 vs 39.9±3.1 g, *P*<0.05), compared with rats fed the HVF diet. Higher visceral adiposity was positively correlated with serum insulin (*r*=0.376, *P*<0.05) and homeostatic model assessment insulin resistance (*r*=0.391, *P*<0.05).

**Conclusion:**

We conclude that lard-based high-fat diets accentuate the increase in weight gain and the development of obesity and insulin resistance more than hydrogenated vegetable-shortening diets. These results further point to the importance of standardizing fatty acid composition and type of fat used in determining outcomes of consuming high-fat diets.

## Introduction

Obesity is associated with increased energy consumption and preference for dietary fat.^1^ In addition, the influence of the type of fat consumed on health has been debated for years and vegetable oil is recommended over animal fats for the reduction of cardiovascular disease and oxidative stress,^2, 3, 4, 5^ which has resulted in an increased intake of omega-6 fatty acids, especially linoleic acid (LA).^6^

To investigate the role of high-fat diets in human obesity, animal models are often utilized.^7^ Many studies in rodents use either animal fat (lard)^8, 9^ or hydrogenated vegetable oil (shortening).^10, 11^ In addition, at least two types of vegetable shortening are used to formulate animal high-fat diets (partially hydrogenated soybean/palm oils or partially hydrogenated soybean/cottonseed oils), which differ greatly in their fatty acid composition. Yet, there are almost no reports of a direct comparison of these commonly used fat sources on the development of diet-induced obesity. Only one study^12^ directly compared growth-promoting properties of lard and vegetable shortening (Crisco) and found no differences in weight gain between these two groups, but neither the type of vegetable shortening nor the fatty acid composition was reported.

Finally, the fatty acid composition of fats used in experimental diets is often based on the USDA tables^13^ rather than being directly measured. A recent report^14^ showed almost twice the amount of LA present in lard than is reported by USDA. Higher LA content in high-fat diets has been linked to hyperphagia, increased adiposity via activation of the brain's endocannabinoid system,^15, 16^ and related insulin resistance,^17^ supporting the need to directly measure fatty acid composition including LA in fat sources to determine their role in the development of obesity.

Therefore, to better understand the functional importance of the source and composition of fat in commonly used experimental high-fat diets, we compared the effects of lard- and vegetable-fat diets on energy intake, body composition and insulin resistance in rats. Adiposity and insulin resistance were the primary dependent measures. Moreover, because lard contains naturally high amounts of vitamin D (USDA nutrient database^13^), vitamin D in the diets and blood was also measured as low vitamin D status has been associated with obesity and characteristics of the metabolic syndrome.^18, 19, 20^

## Materials and methods

### Animals and diets

Male Wistar rats at 21 days of age were purchased from Charles River Inc., (Senneville, Quebec, Canada). Upon arrival, animals were housed individually and maintained on a 12-h light–dark cycle (light on at 0700 h at 22±1 °C) and had *ad libitum* access to water via an automatic water-dispensing system (Allentown Inc., Allentown, NJ, USA). All the procedures were approved by the University of Toronto Institutional Animal Care and Use Committee. Our study was unblinded, randomized and used a sample size of 10 animals per group. A sample size of 10 was required to detect 10% differences in our primary outcome measures (adiposity and insulin resistance) among treatment groups based on *a priori* power analysis (power=0.80). Animals were allocated into three groups using an online randomization tool (www.graphpad.com/quickcalcs), and assigned to receive one of three custom-formulated AIN-93G-based pelleted diets (Dyets, Bethlehem, PA, USA) *ad libitum* for 14 weeks. Group 1 was fed a high-vegetable-fat diet (HVF, 60 kcal% from vegetable shortening); Group 2, a high-lard-fat diet (HLF, 60 kcal% from lard). Group 3, normal-fat (NF, 16 kcal% from vegetable shortening) diet served as ‘negative' control and was primarily used to evaluate the magnitude of the effect(s) owing to the exposure to high-fat diets. The kilocalorie was used as a unit of energy. Vitamins and minerals were added on the basis of the energy density of the diets consistent with that of the AIN93G diet.^21^ Vitamin D amounts in the diets were confirmed by an analytical testing laboratory (Maxxam Analytics, Mississauga, ON, Canada) and were as follows: NF (~22 IU per 100 kcal), HVF (~22 IU per 100 kcal) and HLF (~25 IU per 100 kcal). Diet compositions are presented in Supplementary Table 1. Primex pure vegetable shortening, a mixture of partially hydrogenated soybean and palm oil, was used by Dyets Inc. (Bethlehem, PA, USA) to formulate NF and HVF experimental diets.

The fatty acid composition of each diet (Table 1) was measured by gas chromatography with flame ionization detection as described below. Pelleted diets were ground using a glass mortar and pestle. Approximately 0.3 g of diet was added to 4 ml chloroform and 2 ml methanol and stored at 3 °C overnight. To this, 1.75 ml of 0.88% KCl was added and the mixture was centrifuged and the chloroform was extracted. Another 4 ml of chloroform was added to the methanol and KCl and the chloroform was extracted again and added to the total lipid extract from the previous step. A portion of total lipid extract was transmethylated using 14% boron trifluoride in methanol and fatty acid methyl esters (FAME) were extracted with hexane and quantified using gas chromatography with flame ionization detection as previously described.^22^ Fatty acid methyl esters were analyzed using a Varian-430 gas chromatograph (Varian, Lake Forest, CA, USA) equipped with a Supelco capillary column (SP-2560; 100 m × 0.25 mm inner diameter × 0.20 μm film thickness) and a flame ionization detector. Samples were injected in splitless mode. The injector and detector ports were set at 250 °C. Fatty acid methyl esters were eluted using a temperature program set initially at 60 °C for 2 min, increasing at 10 °C min^−1^ to 170 °C, and held at 170 °C for 4 min, then increasing at 6.5 °C min^−1^ to 175 °C, 2.6 °C min^−1^ to 185 °C and 1.3 °C min^−1^ to 190 °C, increasing at 8 °C and held at 240 °C for 11 min to complete the run at 42.71 min. The carrier gas was helium, set to a constant flow rate of 3 ml min^−1^. Peaks were identified by retention times of authentic fatty acid methyl ester standards (Nu-Chek Prep, Inc., Elysian, MN, USA). The concentration of each fatty acid was expressed as percent of total energy.

### Body weight, energy intake, feed efficiency and LA intake

Body weight was measured from 0900 h to 1000 h once a week for 14 weeks until termination. Food intake (g) was measured once a week and presented as energy intake (kcal per day). The feed efficiency was calculated as a ratio between total body weight gain and cumulative energy intake after 14 weeks on the respective diets.

To better describe the relationship between obesity, insulin resistance and LA content, we calculated estimates of average daily LA intake from the diets; research has demonstrated a positive correlation between LA intake and increased fasting insulin, insulin resistance and adiposity.^17^ Average daily LA intake (mg) was calculated by multiplying average daily energy intake (kcal) by known concentrations of LA in the diets (mg kcal^−1^) and by 0.994 (99.4% is the digestion efficiency of LA in Wistar rats^23^).

### *In vivo* magnetic resonance imaging evaluation of adipose tissue mass

*In vivo* magnetic resonance imaging (MRI) analysis was conducted before animals were killed to measure changes in the subcutaneous and visceral fat compartments. MRI images were acquired at the STTARR Imaging Centre (Toronto, ON, Canada) using a 7 Tesla BioSpec 70/30 USR (Bruker Corporation, Ettlingen, DE, USA), equipped with a B-GA20S gradient coil and 15.5 cm inner diameter quadrature volume coil. Rats were anesthetized and oriented centrally within the radiofrequency coil, and placed in the prone position upon padding. Isoflurane at 1.8% was delivered via nose cone. The slice package consisted of a set of 27 coronal two-dimensional T2-weighted rapid acquisition with relaxation enhancement slices, with 2 mm thickness (54 mm of anterior–posterior coverage). Nine data averages were acquired to reduce respiratory artifacts, resulting in an average acquisition time of 10 min 24 s per animal. MRI data were analyzed using three-dimensional MIPAV (medical image processing, analysis and visualization) software version 7.0.1 (NIH, Bethesda, MD, USA). Visceral and subcutaneous fat pads were segmented semiautomatically by an independent operator on each MR image by selecting a signal intensity threshold value for adipose tissue, followed by manual adjustment. Adipose volume was then converted to grams of fat mass on the assumption of a fat density of 0.9 g cm^−3^,^24^ and presented as a percentage of total body mass on the day of the scan.

### Tissue and serum collection

At the end of week 14, rats were killed by decapitation using a small decapitator guillotine (Harvard Apparatus, Holliston, MA, USA) after a 10 h overnight fast. Blood was collected in serum collection tubes (BD, Franklin Lakes, NJ, USA) and serum was obtained by centrifugation at 1000 *g* for 15 min. Serum aliquots were immediately placed on dry ice and transferred to a −80 °C freezer for later analyses. Because MRI did not allow for accurate measurement of individual compartments of visceral fat, the epididymal, mesenteric and retroperitoneal fat pads were dissected *post-mortem* and weighed. Adipose mass (g) was further adjusted for total body weight and presented as percent body weight.

### Biochemical serum measurements

Fasting glucose concentrations were determined using a one-touch glucometer (Accu-Chek Compact Plus, Roche Diagnostics, Indianapolis, IN, USA). Rat insulin (cat. #EZRMI-13 K, intra-CV: <3% inter-CV: <8%) levels were measured with commercial ELISA kits (Millipore, Billerica, MA, USA). Insulin resistance and insulin sensitivity were evaluated via surrogate indexes: the homeostatic model assessment insulin resistance (HOMA-IR) and the quantitative insulin sensitivity check index (QUICKI). HOMA-IR and QUICKI were calculated from fasting serum glucose (FG, mg dl^−1^) and insulin (FI, μU ml^−1^) using the following formulae:^25^ [HOMA-IR=(FG × FI)÷2430] and QUICKI=1÷[log (FG)+log (FG)]. Vitamin D status was assessed by measuring the 25-hydroxyvitamin D_3_ 25[OH]D_3_ levels in rat serum by electrochemiluminescence immunoassay at the Mount Sinai Hospital (Toronto, ON, Canada) on Roche Modular E170 Analyzer (Roche Diagnostics, Basel, Switzerland).

### Statistical analysis

SAS statistical software (Version 9.3 SAS Institute Inc., Cary, NC, USA) was used for all analyses. Normality of distributions was confirmed by histogram and Kolmogorov–Smirnov test before statistical analysis. Changes in energy intake and body weight over time were analyzed using repeated-measures analysis of variance using Proc Mixed model procedure. When a significant interaction was found, one-way analysis of variance by Proc Glm followed by Tukey's HSD *post hoc* test was used to determine if there were significant differences among treatment groups at each week of the experiment. One-way analysis of variance was also used to test the main effects of experimental diets on body weight, serum insulin, 25[OH]D_3_ and adipose mass. Pearson's correlation coefficients were used to evaluate the associations between adiposity measures and both serum insulin and HOMA-IR. A Bland–Altman analysis was conducted to compare the two experimental methods for determining visceral adiposity: the MRI to the necropsy method. All the data were expressed as mean±s.e.m. Statistical significance was declared at *P*<0.05.

## Results

### Body weight, energy intake, feed efficiency and LA intake

**Figure 1:**
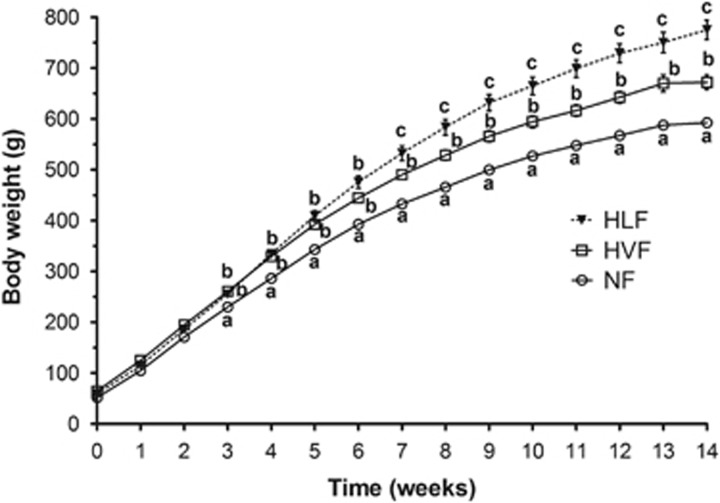
Body weight of rats fed experimental diets over 14 weeks. Different superscript letters identify significant differences between the groups and within each week (*P<*0.05). Values are mean±s.e.m, *n=*10 per group.

A repeated-measures mixed model analysis of variance revealed significant main effects of diets (*P*<0.05), time (*P*<0.05) and a significant interaction (*P*<0.05) on body weight. As shown in Figure 1 throughout the 14-week period, the rats fed both high-fat diets, on average, gained more weight than those fed NF diet. Starting at week 7, body weight was significantly different between all the dietary groups where the pattern observed was as follows: NF<HVF<HLF. The HLF group (532.6±14.3 g) was ~9% heavier than the HVF group (490.4±8.9 g) and ~23% heavier than the NF group (432.9±6.5 g). This difference continued to increase until week 14 where the HLF group (775±19.2 g) had ~15% greater body weight than the HVF group (672.3±14.3, *P*<0.05) and ~31% greater body weight than the NF group (592.5±9.5 g, *P*<0.05).

**Figure 2:**
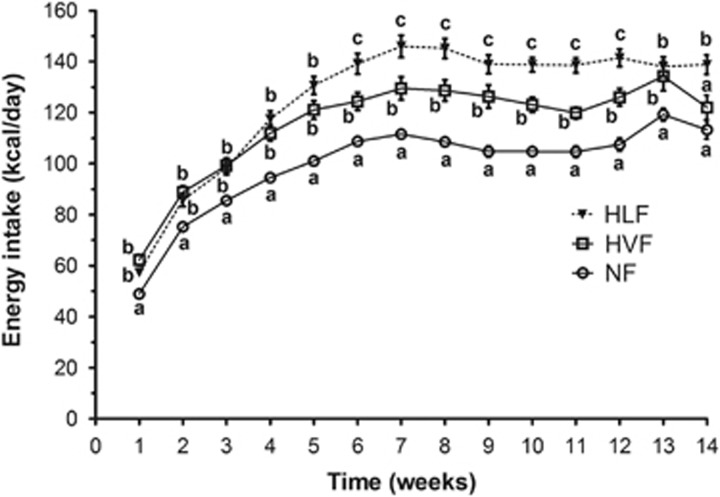
Energy intake of rats fed experimental diets over 14 weeks. Different superscript letters identify significant differences between the groups and within each week (*P<*0.05). Values are mean±s.e.m, *n*=10 per group.

Energy intake was affected by diet (*P*<0.05), time (*P*<0.05) and diet and time (*P*<0.05) interaction (Figure 2). Energy intake followed patterns similar to body weight, where energy intake was highest in rats fed the HLF diet compared with rats fed either the HVF or the NF diet. Furthermore, average energy intake (kcal per day) over the 14-week period also varied significantly between all the groups, where the rats fed the HLF group had the highest energy intake (125.3±2.2) compared with the rats fed both the HVF (115.5±2.2) and the NF diet (99.8±2.2). In addition, cumulative energy intake (kcal) after 14 weeks on diets was also significantly different between all three diet groups. However, rats fed the HLF diet had the highest cumulative caloric intake compared with animals fed either the HVF or the NF diet. Feed efficiency was not different between the NF and HVF groups. There was ~9% higher body weight gain per 100 kcal of diet consumed in the HLF group compared with the HVF group (*P*<0.05; Table 2).

Last, rats in the HLF group consumed on average ~3.2-fold (*P*<0.05) more of LA than those in the HVF group (1556±33 vs 492±13 mg per day, respectively). Average LA intake in the NF group (136±2 mg per day) was significantly lower compared with either of the other two high-fat groups.

### Adiposity

**Figure 3:**
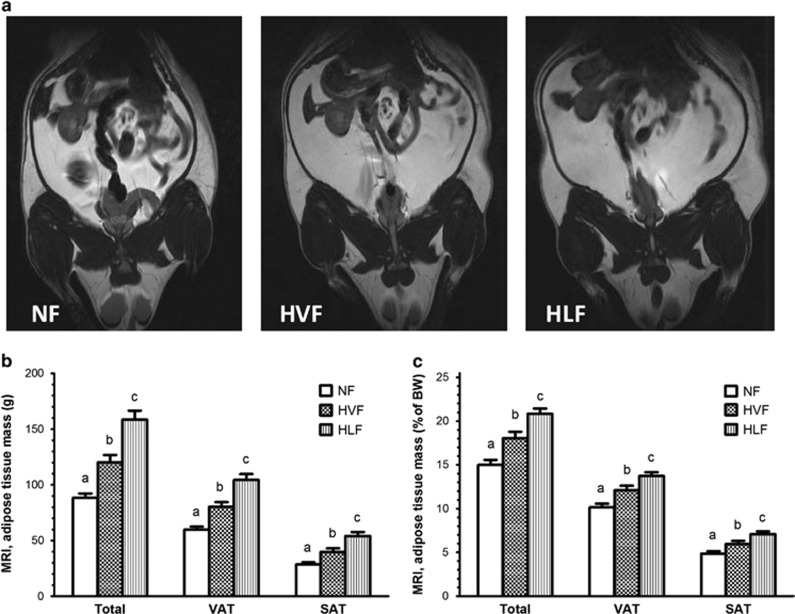
(**a**) Representative MRI images of rats fed experimental diets; (**b**) Total, visceral (VAT) and subcutaneous (SAT) fat mass (g); and (**c**) calculated as percentage of total BW. Different superscript letters identify significant differences between the groups at week 14 (*P<*0.05). Values are mean±s.e.m, *n*=10 per group. BW, body weight.

*In vivo* MRI analysis demonstrated differences in fat mass accretion between the groups. Representative MRI images of rats fed experimental diets, and total, visceral and subcutaneous adipose tissue mass are presented in (Figure 3). As expected, all animals on high-fat diets had greater fat mass than animals on the recommended fat diet. However, rats fed the high-lard-fat diet accumulated the most fat (g) and had ~32% (158.5±8.2 vs 120.2±6.6, *P*<0.05), 30% (104.4±5.2 vs 80.3±4.2, *P*<0.05) and 36% (54.1±3.6 vs 39.9±3.1, *P*<0.05) higher total, visceral and subcutaneous fat mass, respectively, compared with rats fed the high-vegetable-fat diet. There was a similar pattern of change in body composition in response to diets measured by weighing visceral fat pads dissected during necropsy (Table 2). The Bland–Altman analysis was used to compare the mass of adipose tissue (percent body weight) calculated from MRI analyses with the mass measured at necropsy (Supplementary Figure 1). The two methods delivered very similar results, and the bias (difference between the means) was only 0.8. The 95% limits of agreement were between −2.44 to 0.84.

### Serum glucose, insulin and surrogate indices of insulin resistance

Greater visceral adiposity observed in the HLF diet was accompanied by higher circulating insulin levels (Table 3). A positive correlation was found between visceral fat mass and serum insulin (*r*=0.376, *P*<0.05). Serum insulin levels in the HLF group were ~35% higher compared with the HVF group (93.4±6.2 vs 69.1±5.6 μU ml^−1^, *P*<0.05). Serum insulin levels in the HVF and the NF groups were similar even though the former were significantly fatter.

Insulin resistance and insulin sensitivity were evaluated by calculating the HOMA-IR and QUICKI indices. The HLF rats had higher HOMA-IR (4.94±0.35 vs 3.56±0.27, *P*<0.05) and lower QUICKI (0.246±0.002 vs 0.255±0.002, *P*<0.05) than the HVF group; HVF and NF groups were not different. HOMA-IR was positively and significantly correlated with visceral adiposity (*r*=0.391, *P*<0.05).

### Vitamin D status

As a secondary measure, we examined the effects of the experimental diets on vitamin D status. The HLF group had higher levels (44.1±2.9 nmol l^−1^) of 25[OH]D_3_ in serum compared with both the HVF (25.7±2.0 nmol l^−1^, *P*<0.05) and the NF (25.0±1.4 nmol l^−1^, *P*<0.05), respectively, consistent with the presence of vitamin D in lard. Serum 25[OH]D_3_ levels were positively and significantly (*P*<0.05) correlated with serum insulin (*r*=0.356) and HOMA-IR and (*r*=0.418), but not with serum glucose (*r*=0.191, *P*=0.32).

## Discussion

This study shows that the fatty acid composition of a diet impacts energy intake and body weight as well as body composition over a prolonged period in rats. It also demonstrates the importance of elucidating the fatty acid composition in the fat source used in test diets aimed at examining the role of dietary fats as determinants of obesity. Rats fed the HLF diet consumed more calories, gained more weight and body fat than those fed the high-fat diet with vegetable shortening. Rats fed the high-lard-fat diet also developed insulin resistance associated with increased visceral adiposity.

The lard diet was included as a test group for three reasons. First, high-fat diets are based most frequently on either lard or partially hydrogenated vegetable oils. Second, lard contains significant amounts of LA, dependent on the animal feed used to produce lard, which has been associated with hyperphagia and excessive adipose tissue development.^15, 16^ Third, lard is naturally rich in vitamin D, which may act to reduce the metabolic consequences associated with obesity, as suggested by other investigators.^18, 19, 20^

All the animals fed high-fat diets exhibited increased weight gain consistent with higher energy intake. However, the HLF group consumed more calories than the HVF group. There are several possible reasons for this. First, overconsumption in the HLF diet may be owing to the rats' strong preference for the taste of fat sources containing long-chain fatty acids (that is, oleic and linoleic)^26^ as the voluntary food intake was higher in rats fed the HLF diet despite being similar in caloric content to the HVF diet. Second, we found the polyunsaturated content (by weight) of the HLF diet to be 20.2%, 18.8% having been attributed by LA, which is much higher than the USDA database report of 11.2% polyunsaturated fatty acids (LA content was not reported).^13^ This discrepancy between the USDA database and our measured result is important, as it confirms the need to directly measure fatty acid composition in fat sources used in studies looking at the effect(s) of fat intake on obesity. Third, saturated fat in high-fat diets may have also contributed to the increased fat accretion observed in HLF group; however, because the HLF diet was only 3% higher in saturated fats than the HVF diet but threefold higher in LA, and high LA intake has been linked to overeating and obesity,^15, 16^ it is plausible that in our study LA was a greater contributor to the increase in energy intake and adiposity than saturated fats. In addition to greater energy intake, the HLF group had the highest feed efficiency, consistent with previous observations showing that lard is more easily absorbed than palm oil-based vegetable shortening.^27^ The HVF and NF groups had similar feed efficiencies, demonstrating that the greater weight and fat gain in the HVF group was owing to higher energy consumption from the more energy-dense diet.

Difference in fat mass accumulation in response to diets was confirmed by *in vivo* MRI analysis and necropsy and supports other observations^28^ that MRI analysis is an accurate and valid method for monitoring the progression of adiposity *in vivo*. Increased visceral adiposity, particularly mesenteric fat, found in the HLF group was associated with hyperinsulinemia and insulin resistance, which is consistent with other research studies.^29, 30^ Likewise, lard ingestion has been previously shown to independently increase plasma insulin concentrations measured a few hours after ‘lights on' and ‘the last meal of the night' and to associate with increased abdominal adiposity in rats.^31^ Furthermore, we found that HLF group had higher LA intake and higher HOMA-IR, consistent with other reports showing that consumption of diets rich in LA is positively associated with increased fasting insulin, insulin resistance and adiposity.^17^ However, there was no difference in circulating insulin and HOMA-IR between rats that consumed the NF and the HVF diets. These results may suggest that the HVF group did not have sufficient time to accumulate enough visceral fat mass above the threshold at which insulin sensitivity is significantly reduced. This was not the case for the HLF group exhibiting enhanced visceral fat deposition associated with increased energy intake and higher energy retention.

In contrast to previous studies in humans that have associated obesity with decreased bioavailability of vitamin D,^32, 33^ there were no differences in serum vitamin D levels between NF and HVF groups even though body fat was much higher in the HVF group. The latter may suggest that, at least in rats, vitamin D status does not deteriorate with obesity or with high-fat consumption *per se* if vitamin D intake is satisfactory. To provide a definite answer to this question, a measure of vitamin D levels in adipose tissue would be required and is recommended for all the studies assessing vitamin D status in obese subjects. Even though the HLF diet significantly increased vitamin D status in rats, there was no evidence suggesting that the vitamin D content of lard is protective against the development of high-fat diet-induced weight gain or insulin resistance, as shown by other investigators;^18, 19, 20^ it is also plausible that these effects, if any, may be overridden by the influence of lard. Similarly, no effect of vitamin D supplementation on weight reduction^34^ or improvement in cardiovascular disease risk markers (that is, glucose tolerance, blood pressure and serum lipids)^35^ was found in obese and overweight adults. In addition, the HLF diet contained only 14% more of the vitamin D compared with the HVF diet, suggesting that the vitamin D content in lard is less likely to influence weight gain than the fatty acid composition of the diets.

The present study design has some limitations that future research can address. For example, equalizing the LA content in the two test diets would help to further define the role of fatty acid composition. Moreover, even though we examined energy intake and energy retention, reduced energy expenditure may have further contributed to fat accumulation, but was not assessed in this study. In addition, the presence of high levels of trans fats in the HVF diet may have also been a factor that affected food intake. Trans-fat intake is shown to be associated with higher intra-abdominal adiposity and insulin resistance in monkeys fed trans fatty acids (TFAs) for 6 years under a controlled feeding regimen (restricted energy),^36^ possibly explaining increased adiposity in rats fed the HVF diet (containing ~15% of energy as TFAs), but not in the HLF group (containing <1% of supplied energy as TFAs). In contrast, a study in rats showed that dietary TFAs fed *ad libitum* did not influence food intake or body fat accumulation.^37^ Thus the role of TFAs in weight gain and body fat accumulation remains unclear.

The strengths of this study reside in the comprehensive analysis of the fatty acid composition of high-fat diets, which allowed for the identification of the effect of fat source on food intake, body composition and insulin resistance. While the relevance of these findings to humans is uncertain at present, the results show that studies designed to examine the effects of high-fat diets on food intake control and obesity need to be more rigorous in defining the fat source and its composition, both for the purpose of better understanding the mechanism(s) of fat-induced obesity and for the application to dietary recommendation for humans.

We conclude that lard-based high-fat diets accentuate the increase in weight gain and the development of obesity and insulin resistance more than hydrogenated vegetable shortening diets. These results further point to the importance of standardizing fatty acid composition and type of fat used in determining outcomes of consuming high-fat diets.

## Figures and Tables

**Table 1:** Fatty acid composition of experimental diets

*Fatty acid*	*% Total energy*		
	*NF*	*HVF*	*HLF*
14:00	0.11±0.003	0.16±0.01	0.87±0.001
16:00	3.53±0.033	10.74±0.05	14.42±0.07
16:1-9c	0.02±0.001	0.05±0.001	1.09±0.01
18:00	2.20±0.037	9.21±0.25	7.50±0.06
18:1-6t	0.69±0.012	3.13±0.62	ND
18:1-9t	2.30±0.007	10.24±0.64	0.46±0.01
18:1-6c	0.49±0.020	2.36±0.03	ND
18:1-9c	5.14±0.045	16.93±0.36	21.93±0.04
18:1-11c	0.46±0.010	1.92±0.06	1.41±0.01
18:2-9c,12t+19:1-7t	0.13±0.006	0.56±0.01	0.11±0.03
18:2-9t,12c	0.18±0.003	0.78±0.02	0.10±0.02
18:2-9c,12c	1.27±0.025	3.85±0.07	11.37±0.09
18:3-9c,12c,15c	0.06±0.010	0.13±0.03	0.82±0.01
			
Total	16.57±0.08	60.05±1.00	60.08±0.14
Saturated	5.84±0.05	20.11±0.26	22.80±0.09
Unsaturated	7.43±0.05	25.23±0.38	36.61±0.10
Trans-fat	3.30±0.02	14.71±0.89	0.67±0.04
Linoleic acid	1.27±0.03	3.85±0.07	11.37±0.09

Abbreviations: HLF, high-lard-fat; HVF, high-vegetable-fat; ND, concentration was at or below the limit of detection; NF, normal-fat.

Values are means±s.e.m. (*n=*3). Data are expressed as percent of total energy.

**Table 2:** Effects of diets on body weight, energy intake, feed efficiency and adipose tissue mass dissected during necropsy at week 14

*Parameter*	*Group*		
	*NF*	*HVF*	*HLF*
Body weight (g)	592.5±9.5^a^	672.3±14.3^b^	775.0±19.2^c^
Weight gain	540.4±9.1^a^	607.3±14.9^b^	715.8±18.9^c^
Cumulative energy intake (kcal × 10^3^)	9.8±0.1^a^	11.3±0.3^b^	12.3±0.3^c^
FE, weight gain in grams per 100 kcal	5.53±0.1^a^	5.37±0.1^a^	5.83±0.1^b^
Epididymal fat (g)	18.6±1.1^a^	24.8±1.2^b^	30.7±1.0^c^
Epididymal fat (% BW)	3.1±0.2^a^	3.7±0.2^b^	3.9±0.1^b^
Retroperitoneal fat (g)	23.6±1.5^a^	33.6±1.9^b^	45.5±3.0^c^
Retroperitoneal fat (% BW)	4.0±0.2^a^	5.0±0.3^b^	5.8±0.3^b^
Mesenteric fat (g)	14.1±0.8^a^	18.0±1.3^b^	24.5±1.7^c^
Mesenteric fat (% BW)	2.4±0.1^a^	2.6±0.1^a^	3.3±0.1^b^
Total visceral fat (g)	56.3±2.9^a^	76.4±4.2^b^	100.8±4.6^c^
Total visceral fat (% BW)	9.5±0.5^a^	11.3±0.5^b^	13.0±0.5^c^

Abbreviations: BW, body weight; FE, feed efficiency; HLF, high-lard-fat; HVF, high-vegetable-fat; NF, normal fat.

Means within a row with different superscript letters are significantly different (*P*<0.05). Values are means±s.e.m, *n=*10 per group.

**Table 3:** Serum parameters of rats fed experimental diets at week 14

*Parameter*	*Group*		
	*NF*	*HVF*	*HLF*
Glucose (mg dl^−1^)	129±2.8	131±4.0	128±3.1
Insulin (μU ml^−1^)	68.5±5.1^a^	69.1±5.6^a^	93.4±6.2^b^
HOMA-IR	3.66±0.31^a^	3.56±0.27^a^	4.94±0.35^b^
QUICKI	0.255±0.002^a^	0.255±0.002^a^	0.246±0.002^b^

Abbreviations: HLF, high-lard-fat; HOMA-IR, homeostatic model assessment insulin resistance; HVF, high-vegetable-fat; NF, normal fat; QUICKI, quantitative insulin sensitivity check index. Means within a row with different superscript letters are significantly different (*P*<0.05). Values are means±s.e.m, *n=*10 per group.
