# Supplementary material for: A comparison of effects of lard and hydrogenated vegetable shortening on the development of high-fat diet-induced obesity in rats
Source: Nutr Diabetes. 2015 Dec 14;5(12):e188–. doi: 10.1038/nutd.2015.40 (PMC4735054; doi:10.1038/nutd.2015.40)

**Supplementary Figure 1.** Bland–Altman graph displaying differences against average values between two methods for determining of visceral adiposity (VAT) in rats: necropsy and non-invasive Magnetic Resonance Imaging (MRI).


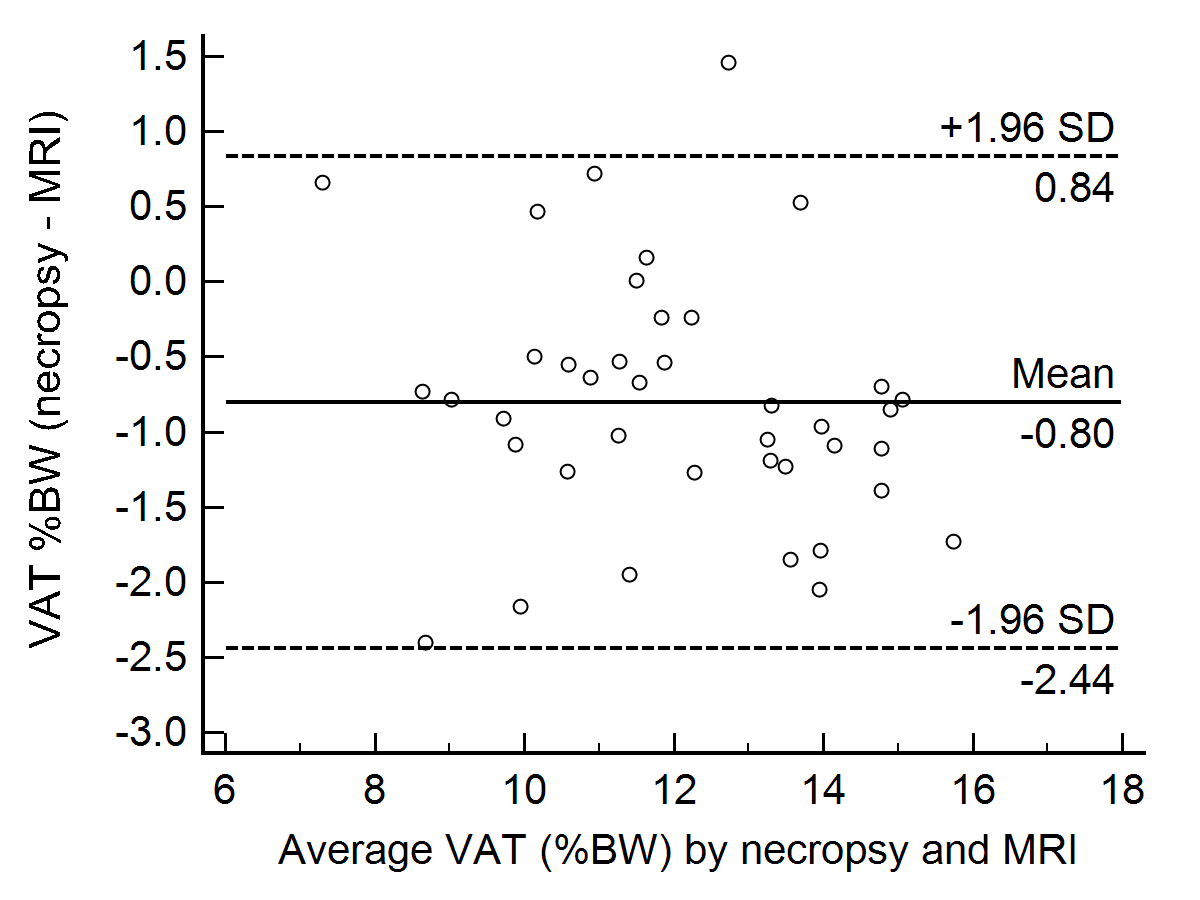

Supplement: Supplementary Figure 1 [file nutd201540x2.doc]
